# Brushing as Environmental Enrichment in Dairy Cattle: Effects of Different Brushing Modalities on Behavior, Health, and Production

**DOI:** 10.3390/vetsci13050450

**Published:** 2026-05-02

**Authors:** Sandra Patricia Maciel-Torres, Alexis Ruiz-González, José Felipe Orzuna-Orzuna, Pablo Arenas-Báez, Jonathan Raúl Garay-Martínez, Lorenzo Danilo Granados-Rivera

**Affiliations:** 1 Unidad Regional Universitaria de Zonas Áridas, Universidad Autónoma Chapingo, Bermejillo 35230, Durango, Mexico; smacielt@chapingo.mx (S.P.M.-T.); jforzuna@gmail.com (J.F.O.-O.); parenasb@chapingo.mx (P.A.-B.); 2 Département des Sols et de Génie Agroalimentaire, Université Laval, Québec City, QC G1V 0A6, Canada; alexis.ruiz-gonzalez.1@ulaval.ca; 3 Campo Experimental Las Huastecas, Instituto Nacional de Investigaciones Forestales, Agrícolas y Pecuarias, Villa Cuauhtémoc 89610, Tamaulipas, Mexico; 4 Campo Experimental General Terán, Instituto Nacional de Investigaciones Forestales, Agrícolas y Pecuarias, General Terán 67400, Nuevo León, Mexico

**Keywords:** dairy cow welfare, grooming device, animal behavior, milk production performance, health indicators

## Abstract

In recent years, brushes have been increasingly implemented on dairy farms to improve animal welfare, health, and productivity. This study presents a narrative review of scientific studies published over the past decade, selected based on their evaluation of brushing effects on behavioral, physiological, and production parameters in dairy cattle. The evidence consistently indicates that access to brushes increases grooming behavior and is associated with reductions in stress-related indicators, supporting improved animal welfare. In addition, brush use has been linked to improved hygiene and may serve as a non-invasive behavioral indicator of health status, as reduced usage has been associated with conditions such as metritis and lameness. In contrast, evidence regarding productive responses remains more variable. Some studies report increases in milk yield and changes in milk composition; however, these effects are not consistently observed and appear to depend on factors such as animal characteristics, management conditions, brush accessibility, and study design. Overall, brushing appears to be a promising enrichment strategy, but further standardized and long-term research is required to better establish its effects on production and reproductive performance.

## Simple Summary

Many dairy farms are adding brushes for cows, but the public may not know why this matters. This review looked at scientific studies from the past ten years to highlight how brushes affect the behavior, health, and milk production of dairy cattle. The evidence shows that cows strongly like to use brushes and spend more time grooming themselves when brushes are available. This helps them stay cleaner, may reduce dirt and parasites on the body, and is linked with calmer behavior and lower signs of stress. Cows that are sick, especially after calving, often use brushes less, so brush use may also help farmers notice health problems earlier. Some studies suggest that cows with regular access to brushes may produce more milk and show changes in milk quality, such as fat and total solids. However, these effects are not consistently observed across all studies. They may be related to greater comfort, improved rest, and overall well-being, although further research is needed to better understand these relationships. In conclusion, providing brushes is a simple and practical way to improve the daily lives of dairy cows while also supporting farm productivity. This can benefit animals, farmers, and consumers alike.

## 1. Introduction

In recent years, improving dairy cow welfare has become a central objective in modern production systems. This shift reflects both ethical considerations and the recognition that animal well-being is closely linked to productivity and system sustainability [1,2,3]. Within this context, environmental enrichment has emerged as a key strategy to promote species-specific behaviors and enhance animals’ ability to cope with environmental challenges [4]. Among the different forms of enrichment, sensory stimulation—particularly tactile enrichment—has received increasing attention due to its direct influence on behavior and physiological responses [5,6].

Sensory stimulation, which includes tactile, auditory, and visual input, constitutes an innovative and underexplored dimension in dairy farms [5]. For example, auditory enrichment, such as classical or instrumental music played at specific tempos and sound levels, has shown promise in reducing stress and promoting relaxation in dairy cows [5]. In dairy cows, music exposure has been linked to lower serum cortisol levels and increased milk production [7]. Similarly, visual stimuli, such as mirrors or conspecific images, have been explored to alleviate the adverse effects of isolation; however, their effectiveness varies depending on the complexity and realism of the stimuli [8].

Among the sensory enrichments studied to date, tactile stimulation using brushes has received increasing interest due to its direct and measurable impact on the well-being of dairy cows [1]. Mechanical brushing mimics the natural grooming behavior of dairy cows and provides physical and psychological benefits [9]. Studies consistently highlight its ability to increase natural behaviors, improve social interaction, and modulate physiological responses to stress [5]. In natural environments, cows use abrasive surfaces such as trees to groom themselves, which is essential for their comfort and health [4]. In intensive farming systems, brushes replicate grooming activity, satisfying this biological need and contributing to well-being [1]. Related to the above, research by McConnachie et al. [10] showed that dairy cows are highly motivated to access mechanical brushes. These findings highlight the intrinsic value of grooming as a behavioral need in dairy cows. Furthermore, brushes also have benefits for physiological and productive parameters. For example, in Holstein heifers, brushes reduce inactivity periods and increase total feeding time [11], which could result in higher milk production. Furthermore, brushes have been associated with indicators of positive emotional states in dairy cows, such as relaxed ear postures, increased tail wagging, and lower heart rate [12]. These physiological responses suggest that brushing has been associated with reduced indicators of stress and may contribute to improved welfare in dairy cows. Furthermore, the adoption of enrichment strategies, such as brushes, represents a paradigm shift in dairy farming that aligns welfare considerations with production objectives.

Although interest in brush-based enrichment has increased in recent years, the available literature remains fragmented, with studies often focusing on isolated outcome domains such as behavior, physiological responses, or production parameters. Previous reviews have primarily addressed environmental enrichment or welfare indicators in a general context, without systematically integrating these domains in relation to brushing as a specific intervention [13,14,15]

Therefore, the contribution of the present review lies in its integrative perspective, which brings together behavioral, health, and productive outcomes associated with brushing, while also considering sources of variability among studies, including differences in brush type, management conditions, and animal characteristics. By synthesizing these domains within a unified framework, this review aims to provide a more comprehensive understanding of the role of brushing in dairy production systems and to identify key gaps for future research.

## 2. Method

The study adapted a checklist from the Preferred Reporting Items for Systematic Reviews and Meta-Analyses (PRISMA) framework described by Page et al. [16] in reference to the PRISMA scheme. The article selection methodology carried out in this study was divided into three stages: (1) identification of selection strategies and criteria for the required publications; (2) conducting a thorough selection process to assess and evaluate the nominated articles; (3) analysis of the information collected to present the resulting findings.

Initially, the search for scientific articles was performed in the following databases: Web of Sciences, Scopus and Science Direct. Boolean operators (AND and OR) were used in the search for articles, and the keyword string syntax used was (“brushing” OR “animal welfare” OR “brushes”) AND (“dairy cows” OR “cattle” OR “dairy industry”) AND (“health and welfare” OR “breeding and ethology”). Following this search, additional studies were identified by reviewing the reference lists of selected publications. To obtain up-to-date information, the range of publication years used in this study was 2015 to 2025, and only publications written in English were considered.

In addition to the keyword-based search strategy, studies were screened based on predefined inclusion criteria. Eligible studies were required to (1) evaluate the effects of brushing on at least one outcome domain (behavioral, physiological, health, or productive parameters), and (2) be conducted in dairy cattle under experimental or observational conditions. No strict filters were applied regarding study design; however, studies lacking primary data (e.g., reviews, conference abstracts, or theoretical papers) were excluded during the screening process.

Studies were not restricted to a specific outcome type, as the objective of this review was to integrate evidence across multiple domains. However, only studies reporting measurable outcomes related to behavior, health, physiology, or productivity were considered eligible. Studies that did not report a measure of variance (e.g., standard deviation, standard error, or confidence intervals) were excluded. This criterion was applied to ensure that the reported results could be interpreted in terms of variability and reliability, thereby supporting a more robust comparison across studies.

**Figure 1:**
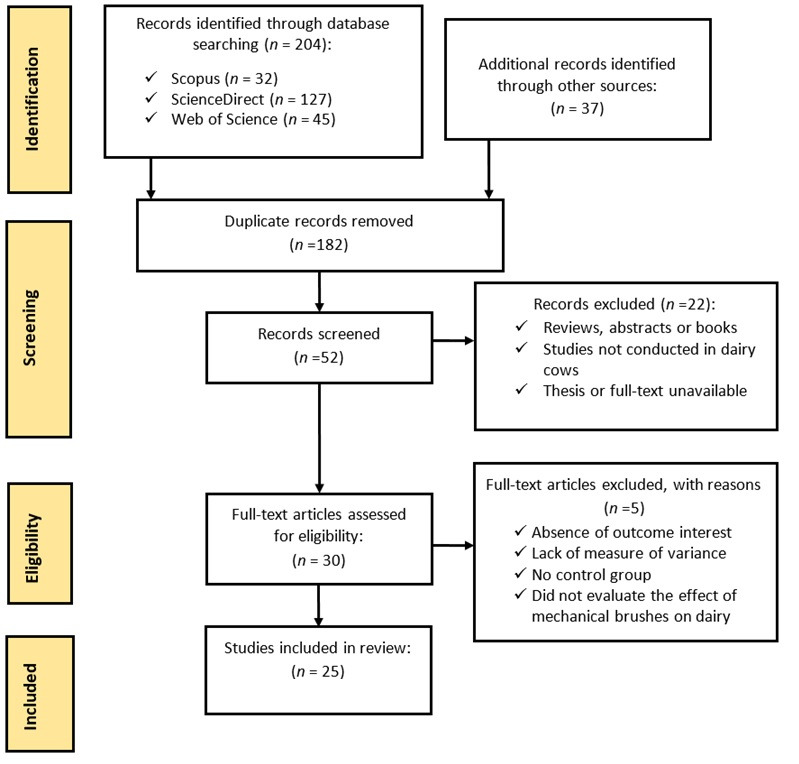
PRISMA flow diagram detailing the literature search strategy and the selection of studies for the literature review.

Figure 1 illustrates the identification and selection process. The selected articles were intended to answer the following questions: What are the effects of brushing on the behavior of dairy cattle? What is the role of brushing in promoting the health and welfare of dairy cattle? How does brushing influence the productive characteristics of dairy cattle?

## 3. The Role of Brushing on the Behavior of Dairy Cattle’s

In dairy cows, the behavioral repertoire encompasses maintenance behaviors (e.g., feeding, rumination, resting) and comfort behaviors (e.g., grooming and scratching, stretching, shaking). In intensive systems, housing design and management are pivotal in determining the time available, and comfort behaviors may be reduced when suitable substrates are lacking. Brushes have been shown to facilitate grooming, especially in hard-to-reach areas of the body, as evidenced in controlled studies on cows [17].

Self-grooming in cattle is characterized by the licking of accessible areas, accompanied by scratching and rubbing against objects to reach the head, neck, back, or rump. The provision of a brush has been demonstrated to modify the distribution of scratching by body region, with an increase in scratching on the neck, back, and tail, and a reduction in scratching of the head. This suggests that substrate substitution may be occurring, and that the behavior is being more readily satisfied [5].

The motivation for the utilization of a resource is indicative of its significance. In a gate-pushing test, cows exerted equivalent effort to access a brush as they did to access fresh feed, suggesting that the brush is a highly valued resource and potentially relevant to their welfare [10,18].

The intensification of dairy farming systems has raised concerns about animal welfare, leading to research into environmental enrichment strategies that promote natural behaviors and reduce animal stress [19]. The use of brushes has emerged as a simple yet effective environmental enrichment strategy that mimics natural grooming behavior, improves hygiene, and increases productivity in dairy cows [20]. Furthermore, the frequency of mechanical brush use can serve as a non-invasive indicator of dairy cow health status, reflecting alterations in activity patterns that may signal deviations from normal behavioral baselines [21,22].

The dairy cows with access to brushes significantly increase their grooming activity and spend more time grooming with the brush than without one [5]. As a starting point, we consider that brushing satisfies the intrinsic motivation to groom and serves as a welfare promoter in dairy cows. Recently, Lecorps et al. [23] reported that Holstein-Friesian dairy cows prefer mechanical brushes to static ones, with peak use during heat stress, suggesting thermoregulatory benefits. This result addresses the motivational driver’s gap, showing that the need for grooming extends beyond maintaining coat health. Nevertheless, Foris et al. [24] revealed that social hierarchy influences access to mechanical brushes, with dominant individuals monopolizing and displacing subordinates, suggesting equity issues among dairy cows.

On the other hand, the utilization of a mechanical brush has been demonstrated to significantly reduce stereotypical repetitive behaviors and indications of boredom or stress in dairy cows [25]. The implementation of an escape route for grooming has been demonstrated to reduce inactive time and chewing on enclosures, thereby allowing dairy cows to engage in normal activities such as feed intake for a greater proportion of their time.

Brush grooming also positively affects social interactions among dairy cows by improving comfort and reducing stress [1]. Dairy cows with brush-enriched environments are generally less agitated, which decreases aggressive encounters [5]. Dairy cows frequently organize themselves neatly to use a shared mechanical brush, demonstrating orderly social use rather than competition [5]. Overall, environmental enrichment with brushes fosters a more stable social environment where dairy cows are more likely to engage in orderly social grooming or rest near other cows without conflict. This is consistent with Sadrzadeh et al. [9], who indicate that environmental enrichment with brushes can improve social harmony in group-housed cattle by reducing frustration and boredom.

Conversely, other studies have posited that the hierarchical structure represents a pivotal factor in determining access to brushing devices and the interpretation of brush-use behavior. As indicated by previous studies, dominant animals have been found to utilize brushes with greater frequency. In contrast, subordinate individuals may exhibit a reduced propensity for brush usage, potentially as a result of either avoidance or displacement, even in the absence of direct aggression [18,24]. This finding indicates that patterns of brush usage are not solely determined by intrinsic motivation but are also influenced by social dynamics within the group.

Importantly, this has direct implications for the interpretation of brush use as a behavioral indicator. A reduction in brush use should not be interpreted exclusively as a sign of compromised welfare or disease, as it may also reflect restricted access due to social constraints. In group-housed systems, subordinate animals may have limited opportunities to interact with brushes, particularly when brush availability is low or placement favors dominant individuals.

From a management perspective, these findings highlight the importance of considering brush-to-cow ratio, spatial distribution, and placement within the housing system to ensure equitable access. Providing multiple brushes or positioning them in less competitive areas may reduce dominance-related exclusion and improve the reliability of brush-use data as an animal-based measure.

Overall, the influence of social hierarchy underscores the need to interpret brush-use behavior within its social context, particularly when it is used as a proxy for health or welfare status.

Alterations in dairy cows’ time budget indicate the impact of brushing on their well-being. Brushing increases low-intensity behaviors (grooming, resting) and decreases walking time, improving relaxation. Brushing modifies behavior and is associated with endocrine responses reflecting changes in affective state and stress regulation. For example, brushing directly modulates the stress axis by promoting relaxation and grooming behavior in cows, leading to reduced cortisol levels [26]. Another study reported lower serum cortisol in cows that received gentle brushing during milking [27]. This reduction in cortisol reflects activation of neurohormonal relaxation pathways.

Tactile stimulation, such as grooming, is known to activate the parasympathetic nervous system and can stimulate the release of oxytocin in the brain, a hormone that induces calming and anti-stress effects (such as reduction in cortisol, heart rate, and blood pressure) [5,28]. Indeed, cows in enriched environments with brushes have lower heart rates and higher heart rate variability, classic signs of reduced stress, and increased parasympathetic tone [29].

The pleasurable nature of brushing likely activates the brain’s reward circuits (dopaminergic pathways) in dairy cows, as the elevated motivation to access brushes suggests that cows experience brushing as rewarding [5]. This effect is typically mediated by the release of dopamine in the brain’s reward centers. Although dopamine has not been directly measured in the cited studies, dairy cows’ effort to access brushes, similar to their effort to access feed troughs, could imply strong positive feedback [10].

Dairy cows groomed with brushes are often described as calmer, with slow tail-wagging behaviors, squinting eyes, and relaxed ear postures during grooming, symptoms consistent with a state of satisfaction and low stress [5]. Likewise, it is possible to assume that the integration of behavioral, health, and production domains indicates that brushing acts through interconnected mechanisms. For example, (1) Behavioral well-being: satisfying grooming urges reduces stress behaviors and promotes natural time budgets [3,23]; (2) Physiological health: improved hygiene and blood flow, together with neuroendocrine modulation, promote immune function and disease resistance [22] and (3) Improved productivity: reduced stress and improved feed digestion translate into improvements in productive performance and milk quality [29].

**Figure 2:**
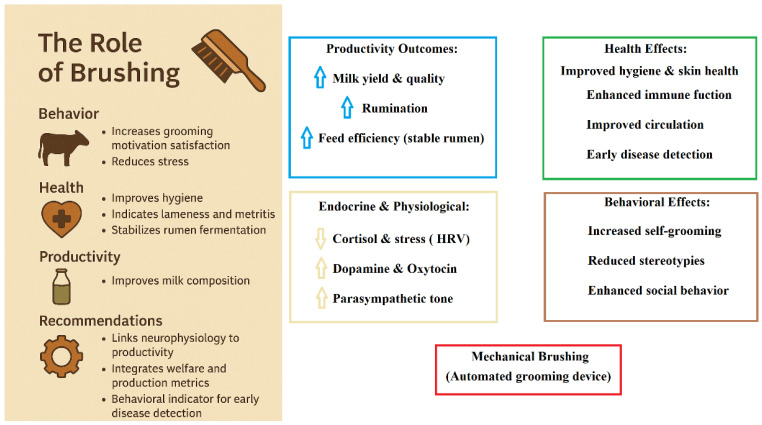
Conceptual model illustrating the proposed pathways linking brushing to behavioral, physiological, and productive outcomes in dairy cows.

Figure 2 summarizes hypothesized relationships derived from the literature reviewed in this study. Brushing, as a form of tactile environmental enrichment, is associated with changes in grooming behavior, time budgets, and social interactions. These behavioral responses may be linked to physiological processes, including modulation of stress-related indicators (e.g., cortisol) and autonomic regulation, as reported in previous studies.

Improved grooming activity may also contribute to better hygiene and integument condition, which have been associated with reduced risk of certain health disorders. In turn, these behavioral and physiological changes may influence productive parameters such as milk yield and composition. Additionally, variations in brush use have been reported as potential indicators of health status in dairy cows.

It should be noted that the pathways presented in Figure 2 are conceptual and based on associations reported in the literature, rather than direct mechanistic evidence. Arrows indicate proposed relationships that may vary depending on management conditions, animal characteristics, and study design [1,5,10,14,27].

Continuing on this topic, housing system is a key moderator, as cows in more restrictive environments (e.g., high-density indoor systems) may exhibit stronger behavioral responses to brushing due to limited opportunities for natural grooming. In contrast, animals in pasture-based or low-density systems may show lower relative engagement with brushes, as alternative grooming substrates are available. Similarly, brush placement within the housing environment plays a critical role in determining usage patterns. Brushes located in high-traffic areas such as feeding alleys tend to be used more frequently, whereas suboptimal placement may limit access and reduce their effectiveness [25].

Group size and brush-to-cow ratio also influence behavioral outcomes. In larger groups or systems with limited brush availability, competition for access may arise, often mediated by social hierarchy. Dominant individuals may use brushes more frequently, while subordinate animals may reduce usage due to avoidance rather than lack of motivation [19,22]. This can lead to an underestimation of the true behavioral demand for grooming within the group.

Taken together, these findings suggest that while brushes generally promote natural grooming behavior and reduce stress-related activities, their effectiveness depends on management conditions that ensure equitable access and adequate stimulation. Therefore, behavioral outcomes should be interpreted within the context of housing design, resource distribution, and social dynamics.

To improve conceptual clarity, it is important to distinguish between behavioral responses and downstream health outcomes. In this review, behavioral changes (e.g., grooming activity, time budgets, and social interactions) are interpreted as primary responses to brush provision, whereas health-related indicators (e.g., disease detection, integument condition, and physiological stress markers) are considered secondary outcomes that may arise through behavioral and physiological pathways. This distinction allows for a more structured interpretation of brushing effects, linking mechanisms to measurable welfare and production consequences.

### Sources of Heterogeneity in Brushing Outcomes

Although the literature consistently reports positive effects of brushing on dairy cow behavior, health, and productivity, considerable variability exists across studies. This heterogeneity is largely driven by differences in brush design, placement, animal density, and patterns of use, which can influence both access to the resource and the magnitude of the observed responses.

One key factor is brush type and functionality. Rotating (mechanical) brushes generally elicit higher usage frequencies and longer interaction times compared to stationary brushes, likely due to increased tactile stimulation and responsiveness to animal contact [23,24]. In contrast, fixed brushes may provide more limited stimulation, potentially reducing their effectiveness as enrichment devices under certain conditions.

Brush placement within the housing system is another critical determinant. Brushes located near high-traffic areas such as feeding alleys or water points tend to be used more frequently, whereas poorly positioned brushes may limit access, particularly for subordinate animals [24]. This effect is closely linked to social hierarchy, as dominant cows may monopolize preferred locations, thereby influencing the distribution of brush use within the group [18].

Herd density and brush-to-cow ratio further contribute to variability in outcomes. Studies have shown that insufficient availability of brushes relative to group size can increase competition and reduce access for subordinate individuals, potentially attenuating welfare benefits [21]. Conversely, providing multiple brushes or optimizing brush-to-cow ratios may promote more equitable use and enhance overall group welfare.

In addition, differences in management and use protocols—such as continuous versus restricted access, integration with milking routines, or human-delivered brushing—can lead to divergent physiological and behavioral responses. For example, tactile stimulation during milking may primarily influence acute stress responses, whereas continuous access to mechanical brushes may have cumulative effects on time budgets and long-term welfare indicators [27].

Taken together, these findings highlight that the effects of brushing are context-dependent and mediated by multiple interacting factors. Therefore, inconsistencies among studies should not be interpreted solely as contradictory evidence but rather as reflecting differences in experimental design and management conditions. Future research should prioritize standardized reporting of these variables to improve comparability and facilitate meta-analytical approaches.

## 4. The Role of Mechanical Brushing in the Health and Well-Being of Dairy Cows

While Section 2 focuses on behavioral responses as immediate indicators of animal-environment interaction, this section examines how these behavioral changes translate into measurable health and welfare outcomes. In particular, brushing may influence health through multiple mechanisms, including improved hygiene, modulation of stress physiology, and its utility as a behavioral indicator of disease. Therefore, this section emphasizes animal-based health indicators and their interpretation within established welfare assessment frameworks.

From a mechanistic perspective, brushing can be understood as a tactile enrichment that acts through three primary pathways: (1) behavioral regulation, by satisfying grooming motivation and reducing frustration; (2) physiological modulation, through effects on the hypothalamic–pituitary–adrenal axis and autonomic balance; and (3) hygiene-related processes, by improving integument condition and reducing pathogen load. These mechanisms collectively contribute to measurable health outcomes, including changes in disease incidence, stress biomarkers, and animal-based welfare indicators [30].

In the field of dairy production, the emphasis on outcomes and prevention is of paramount importance. At the international level, the World Organisation for Animal Health (WOAH) Terrestrial Code for dairy systems underscores the necessity of preventing and controlling conditions that impair animal welfare. Such conditions include mastitis, lameness, metabolic and reproductive diseases. The code further emphasizes the importance of monitoring and management to optimize health and behavior [31]. In the European context, the European Food Safety Authority (EFSA) scientific opinion on dairy cow welfare synthesizes evidence and risks by housing system and emphasizes the use of indicators linked to welfare consequences [32].

With reference to brush studies, priority Animal-Based Measures (ABMs) encompass the following: integument integrity (lesions, alopecia, and inflammation), cleanliness/hygiene, lameness, and behavioral measures of comfort (brush use, rest time). Brushes are classified as a resource-based intervention; however, their evaluation must be based on measurable changes in the animal.

Stress in dairy cows primarily involves the HPA axis (cortisol) and the sympathetic nervous system. The interpretation of these results is contingent upon the time horizon under consideration. Specifically, the analysis of cortisol in saliva or plasma provides a rapid means of detecting changes, whereas matrices such as hair have the capacity to record cumulative exposure over time. In the context of brush studies, it is recommended to interpret physiology as convergent evidence alongside behavior and health, given that the relationship between brush use and cortisol may be weak or dependent on the time of measurement [33].

Cortisol (saliva/milk/hair), heart rate and variability (HR/HRV), infrared thermography, and inflammatory indicators can provide complementary information. However, all are sensitive to confounding factors (time of day, weather, stage of lactation, milking routine, disease), so they must be standardized and analyzed with covariates.

Brushing is a critical indicator of health and well-being in dairy cattle. Grooming behavior is expected to decrease in cows experiencing stress, illness, or energy restriction [14,15,16,17]. Automated systems that monitor brush use have demonstrated the potential to detect health problems such as metritis before clinical symptoms are evident [14] (Table 1). A link has been found between using brushes less and metritis in dairy cows. Cows with metritis use brushes 50% less than healthy cows after giving birth [14]. It has also been reported that dairy cows change their behavior to cope with infections [15]. Behaviors like lethargy, drowsiness and depression help cows save energy (and lose less heat), which helps them cope with the cost of fever. So it is no surprise that cows put saving their own lives before activities that improve their fitness (like using the brush) [14].

Within a precision livestock farming framework, the integration of brush-use monitoring with other sensor-based technologies offers promising opportunities for early disease detection and individualized herd management. Behavioral data derived from automated systems can provide continuous, real-time insights into animal status, but their interpretation should account for individual variability in behavioral responses.

Because responses to technology and novelty differ among individuals, future studies on brush-use monitoring should also consider animal coping style and personality traits. For example, proactive animals have been shown to exhibit calmer responses and faster habituation to automated systems such as milking environments, which may influence their interaction with enrichment devices and sensor-based monitoring tools [35,36,37]. Incorporating these behavioral traits into decision-support systems could improve the accuracy and applicability of behavioral indicators in dairy management.

This perspective highlights the importance of integrating behavioral phenotyping with technological approaches to enhance the precision and reliability of welfare and health monitoring systems.

Brushing influences physiological health through improved hygiene, stress reduction, and disease detection. Cleaner cows are also at lower risk of diseases such as mastitis, as less dirt on the coat and udder decreases the likelihood of bacteria contaminating the teats. Li et al. [1] and Zhang and Li [38] reported that brushing resulted in greater body cleanliness and peripheral blood circulation throughout all stages of lactation. In another study, Moncada et al. [34] observed that dairy cows affected by chorioptic mange initially increased their use of brushes to relieve pruritus and decreased their use after treatment, highlighting brushes as an indicator of skin health. Decreased brushing is common in dairy cows with metritis [14], suggesting that the frequency of brushing could be used as an indicator of reproductive health in dairy cows. Similarly, Burton and Blackie [39] linked reduced brushing use with lameness severity in housed dairy cows, reinforcing its application in mobility monitoring. Furthermore, Velasquez-Munoz et al. [11] found that brushing reduced inactivity and increased feeding time in dairy calves. These health-focused studies demonstrate the potential of brushing as an indicator of health status. However, they often lack long-term follow-up and standardized cleaning score metrics, which limits comparability.

The use of brush visit frequently to detect disease early is based on cows’ prioritization of brushing over basic behaviors such as feeding and rumination [5]. Low-resistance behaviors decline sooner and recover more slowly after treatment [23], providing a nuanced measure of both disease onset and recovery phases.

In this regard, a particularly relevant and emerging aspect of brush use is its potential application as a non-invasive behavioral indicator of health status in dairy cows. It is important to note that behavioral changes can occur before obvious clinical signs appear, suggesting that brush use could serve as an early warning of health problems [14,15,34,35].

From a practical perspective, this establishes brushing as not only an environmental enrichment tool, but also a component of precision livestock management systems.

Automated monitoring of brush use via sensors or integrated farm management technologies could provide continuous, real-time data on individual animal behavior, enabling the early detection of deviations from normal patterns. Such deviations may reflect underlying physiological or pathological changes, offering livestock producers a valuable tool for timely intervention and improved herd management.

However, it is important to note that changes in brush use are not disease-specific and can be influenced by multiple factors, including social hierarchy, environmental conditions and management practices. Therefore, grooming activity should be interpreted alongside other behavioral and physiological indicators to improve accuracy and reliability. Further research is needed to validate the thresholds, sensitivity, and specificity of grooming as a diagnostic support tool under commercial conditions.

## 5. The Role of Brushing on Productive Parameters of Dairy Cows

In dairy production systems, the impact of welfare-oriented interventions such as brushing is typically mediated through indirect pathways. Improvements in comfort behavior, hygiene, and stress reduction may influence metabolic efficiency, endocrine function, and ultimately productive performance. However, compared with behavioral and health outcomes, the evidence base linking brushing to production parameters remains more limited and heterogeneous, with fewer studies explicitly addressing milk composition and reproductive performance.

It has been demonstrated that an improvement in the quality of life, as well as greater stability in health, can result in an enhancement in milk production. Recent studies have indicated that cows provided with access to brushes have exhibited increases in milk production [1]. Additionally, changes in indicators associated with postpartum stress and progesterone following artificial insemination in pregnant cows have been reported. Conversely, milk somatic cell count/raw milk somatic Cell (SCC/RCS) is known to be a more variable outcome, largely determined by intramammary infections and milking and hygiene practices. Consequently, the brush could affect SCC primarily if it leads to improvements in mediators such as hygiene and health.

Improving animal welfare is considered a determining productivity index in modern dairy production systems. Among the various strategies currently available, environmental enrichment with brushes has emerged as a promising tool to improve the productivity of dairy cows [1]. These studies provide strong evidence that brushing contributes to improved performance through behavioral, hormonal, and digestive mechanisms. However, there are discrepancies regarding the direct effects on milk production and the duration of the observed benefits, underscoring the need for long-term, breed-specific, and mechanistic research.

Despite these promising findings, there remains a clear imbalance in the literature, with significantly fewer studies addressing productive and reproductive outcomes compared to behavioral and health parameters. This limitation reflects both the complexity of isolating the effects of brushing from other management factors and the longer time scales required to evaluate production and fertility responses. As a result, current evidence should be interpreted cautiously, and further research using standardized experimental designs, larger sample sizes, and long-term monitoring is needed to establish causal relationships. This constitutes a significant gap in the extant literature. In the study conducted by dos Santos et al. [29], which included 40 Jersey-Holstein crossbred dairy cows aged 36 to 42 months and weighing between 350 and 400 kg, with an average of 162 ± 63.12 days of lactation and a milk yield of 7.4 ± 2.7 L/day, it was demonstrated that access to brushes resulted in a significant increase in daily milk production, with an average increase of 5.24 L per day, compared to 3.49 L per day without brushing. This emphasizes the importance of providing environmental enrichment, such as brushes, for mid-lactation cows. Furthermore, the residual milk was evaluated using oxytocin. It was observed that in bovines with access to brushes, residual milk was lower than in those without access to them.

In a complementary study, Wredle et al. [27] analyzed whether the administration of concentrates or abdominal brushing during mechanical milking modifies the acute release of oxytocin and cortisol. The conclusion of this research was that feed intake improves oxytocin-mediated milk let-down, while gentle brushing reduces cortisol levels, an indicator of stress, without affecting oxytocin. These findings suggest that visceral (feeding) and tactile (brushing) stimuli activate different neuroendocrine pathways. Consequently, the combined incorporation of these elements could synergistically improve productivity and well-being. The integration of physiological biomarkers with conventional behavioral parameters facilitates a comprehensive assessment of dairy cow well-being [5].

Beyond milk yield, a limited number of studies have evaluated the effects of brushing on milk composition. Available evidence suggests that improvements in welfare and reductions in stress may positively influence milk constituents such as fat and total solids, likely through enhanced rumination, more stable feeding patterns, and improved metabolic status. For example, studies incorporating tactile stimulation during milking or daily management have reported increases in milk fat concentration and total solids, which are commonly associated with improved rumen function and reduced physiological stress [29]. Nevertheless, these findings remain inconsistent across studies, and the extent to which brushing alone—independent of other enrichment or management factors—affects milk composition requires further investigation.

Li et al. [1] conducted a study to investigate the impact of rotating brushes on a commercial herd of 522 Holstein cows during the lactation, dry and peripartum phases. The findings of the study demonstrated that there was a 2.3 tons (t) increase in cumulative production (305 days in milk) in fourth-parity cows and a 1.0 t increase in production in fifth-parity cows, with no significant changes observed in second- and third-parity cows. This finding indicates that the productive response is contingent on the number of lactations. With regard to reproduction, the effects were more limited: cows in the brushing group showed higher levels of oestradiol on the day of insemination and numerically greater uterine involution (70% vs. 55%), although further studies are needed to confirm these results.

The effects of brushing on reproductive performance are even less well established. Some evidence suggests that reduced stress and improved welfare conditions may support reproductive physiology through modulation of endocrine pathways, including cortisol and reproductive hormones. For instance, increases in oestradiol concentrations at the time of insemination and improved uterine involution have been reported in cows with access to brushes [1]. However, these responses are not consistently observed, and the current literature lacks sufficient longitudinal and controlled studies to determine whether brushing has a direct and reproducible effect on fertility parameters such as conception rate, calving interval, or days open.

The extant evidence suggests that regular brushing of cows at certain parities improves milk production and contributes to the reduction in stress markers, which in turn promotes cow welfare. In this regard, Table 2 offers a structured overview of the literature. It summarizes the main findings in the behavioral, physiological, health and productivity domains. The summary highlights the differences in the strength and consistency of the evidence across the various outcome categories. The evidence is most robust for behavioral outcomes, which are consistently reported across studies. Indicators pertaining to physiology or health also demonstrate relatively consistent associations, albeit influenced by methodological and environmental factors. In contrast, productive responses remain more variable and context-dependent, reflecting both biological complexity and limitations in the current evidence base.

On the other hand, the reproductive responses of these subjects have yet to be elucidated. Future research should adopt longer-term designs with larger sample sizes, integrating physiological and behavioral measures. This would facilitate a comprehensive understanding of the role of brushing in the health, productivity and reproduction of dairy herds.

## 6. Interpretation of Inconsistent Findings Across Studies

Although the literature generally supports positive effects of brushing on dairy cow welfare and performance, inconsistencies have been reported across studies, particularly regarding productive outcomes. For example, some studies have observed greater benefits in high-producing cows, whereas others report more pronounced effects in multiparous animals [1,29]. These discrepancies likely reflect the interaction of biological, environmental, and methodological factors rather than contradictory evidence per se.

From a biological perspective, parity and production level may influence responsiveness to brushing. Multiparous or high-yielding cows are typically subject to greater metabolic demands and physiological stress, which may increase their sensitivity to environmental enrichment. Consequently, these animals may exhibit more pronounced behavioral and physiological responses to brushing compared to primiparous or lower-producing individuals.

Management-related factors also play a critical role. Differences in housing systems, herd density, and access to resources can influence baseline stress levels and behavioral expression. For instance, in high-density or competitive environments, the benefits of brushing may be attenuated due to limited access or social hierarchy effects, whereas in well-managed systems with adequate brush availability, responses may be more consistent across animals.

Brush-related characteristics, including type (rotating vs. stationary), placement, and accessibility, further contribute to variability. Rotating brushes typically elicit greater engagement, which may enhance their effects on behavior and downstream outcomes. In contrast, suboptimal placement or limited availability may reduce usage and, consequently, the magnitude of observed benefits.

Methodological differences among studies also warrant consideration. Variations in sample size, experimental duration, statistical power, and the selection of outcome variables can influence the detection and interpretation of effects. Studies with small sample sizes or short observation periods may fail to detect significant differences, particularly for complex traits such as milk production or reproductive performance. Additionally, differences in statistical models and covariate control (e.g., stage of lactation, health status) may contribute to divergent conclusions.

Taken together, these factors suggest that variability in reported outcomes is multifactorial and context-dependent. Future studies should aim to standardize experimental conditions and report key variables in detail to facilitate cross-study comparisons and improve the robustness of conclusions.

Despite evidence supporting the use of brushing as an environmental strategy, methodological limitations constrain the strength and generalizability of findings. Many studies have small sample sizes or single-herd designs, limiting statistical power and external validity. Most experiments are short, focusing on acute or short-term effects, restricting the ability to evaluate long-term effects.

Variability in experimental design poses challenges for interpreting results. In some studies, control animals are deprived of enrichment, in others they are exposed to different environmental conditions, making direct comparisons difficult. Inconsistent reporting of variables such as brush type, placement, brush-to-cow ratio, and management conditions limits comparability.

Many studies rely on single-domain indicators, assessing outcomes independently rather than through integrative frameworks. This may obscure causal relationships. Potential confounding factors are not always controlled or reported.

Taken together, these limitations highlight the need for more robust and standardized experimental designs, including larger multi-herd studies, longer monitoring periods, clearly defined control conditions, and the integration of behavioral, physiological, and production metrics. Addressing these gaps will be essential for advancing the scientific understanding and practical implementation of brushing in dairy systems.

## 7. Future Perspective

From a future perspective, the integration of brush-use monitoring into precision livestock farming systems offers opportunities to advance both welfare assessment and herd management. In this context, it is important to consider that behavioral responses to technology and novelty differ among individual animals. Variability in coping style and personality traits may influence how cows interact with enrichment devices and automated monitoring systems, thereby affecting the interpretation of behavioral data.

For example, proactive animals have been shown to exhibit calmer responses and faster habituation to automated dairy technologies, such as milking systems [34,37,39]. This suggests that individual behavioral phenotypes may modulate engagement with brushing devices and the resulting data streams used for health and welfare monitoring. Incorporating these traits into decision-support systems could enhance the sensitivity and specificity of behavioral indicators, including brush-use patterns, for early disease detection.

Overall, this approach highlights the potential of combining behavioral phenotyping with sensor-based technologies to improve the precision and reliability of animal-based measures in dairy production systems.

## 8. Conclusions

A growing body of empirical evidence indicates that brushing appears to satisfy natural grooming behavior and has been associated with reduced stress-related behaviors, improved hygiene, and potential benefits for skin- and udder-related health. These welfare improvements may be accompanied by changes in milk yield and quality, although results are not consistent across studies. Nevertheless, the variability in research methodologies, herd management systems, and socio-economic contexts highlights the need for a more standardized and context-specific approach to evaluating and implementing brushing interventions.

To fully realize the potential benefits of brushes as a welfare-enhancing and productivity-supporting tool, future studies should adopt integrative methodologies that combine behavioral observation, health monitoring, and production metrics. The incorporation of precision livestock technologies—such as automated brush usage tracking, activity sensors, and real-time health indicators—can enable more nuanced assessments and promote evidence-based refinement of brushing protocols. Ultimately, positioning brushing as a routine component of animal enrichment and management strategies may contribute meaningfully to the sustainability and ethical standards of modern dairy production systems.

## Figures and Tables

**Table 1:** Main health and welfare benefits of brush use in dairy cows reported in the literature.

Benefit	Reference
**Useful indicator of emotional state after parturition due to reduced usage:** Cows used brushes 47.5% less during the immediate postpartum period compared to prepartum levels. Cows separated from their calves also used the brush less (8.51%) after giving birth. So, cows might be experiencing anhedonia. This could help with research on the mood of dairy cows.	[22]
**Improving the body cleanliness of dairy cows:** The study found that cows with access to brushes had an average of more than 30% of their body surface area that was clean, in comparison to the body surface area of cows that did not have access to brushes.	[1]
**Relief-seeking behavior during skin disease (chorioptic mange):** Brush use in cows with chorioptic mange decreased by 9.21% during the 10–18 days following treatment. This suggests that brush use in cows with mange decreases after initial use, possibly due to the elimination of mange, and that access to a grooming device is a valuable resource for dairy cows.	[34]
**Reduced use of brushes as an indicator of lameness:** Studies showed cows with lameness used brushes 30% less, especially those far from the trough, but still used those next to it. This suggests that monitoring brush use could detect lameness in dairy cows.	[15,33]
**Early detection of metritis by reducing brushing with mechanical brushes:** Cows diagnosed with metritis showed a 23.43% reduction in brushing frequency during the early postpartum period compared to healthy controls. Similarly, cows diagnosed with metritis brushed for 50% less time than control cows 8–28 days after calving. These results suggest that monitoring the use of brushes on farms could help to detect welfare issues. It can be assumed that slow recovery in the use of brushes after medical treatment could indicate disease recovery.	[14]

**Table 2:** Summary of reported effects of brushing in dairy cattle across outcome domains.

Outcome Domain	Main Findings	Consistency of Evidence	References
Behavior	Increased grooming activity; higher motivation to access brushes; reduction in abnormal behaviors; improved time budgets	Consistent	[5,10,17,23,25]
Physiology	Reduced cortisol levels; increased parasympathetic activity; possible oxytocin-mediated effects	Moderately consistent (context-dependent)	[26,27,28,33]
Health	Improved hygiene; reduced parasite load; reduced brush use associated with metritis, lameness, and skin disorders	Consistent for associations (not disease-specific)	[14,15,35,39]
Productivity	Increased milk yield in some studies; possible improvements in fat and total solids; variable reproductive effects	Inconsistent/limited evidence	[1,27,29]

## Data Availability

No new data were created or analyzed in this study.
